# Novel Gene Rearrangements in the Mitochondrial Genomes of Cynipoid Wasps (Hymenoptera: Cynipoidea)

**DOI:** 10.3390/genes13050914

**Published:** 2022-05-20

**Authors:** Xiaohan Shu, Zekai Li, Ruizhong Yuan, Pu Tang, Xuexin Chen

**Affiliations:** 1 Hainan Institute, Zhejiang University, Sanya 572000, China; xhshu@zju.edu.cn (X.S.); xxchen@zju.edu.cn (X.C.); 2 Guangdong Laboratory for Lingnan Modern Agriculture, Guangzhou 510642, China; 3 State Key Laboratory of Rice Biology, Zhejiang University, Hangzhou 310058, China; 21816088@zju.edu.cn (Z.L.); monareason@zju.edu.cn (R.Y.); 4 Institute of Insect Sciences, College of Agriculture and Biotechnology, Zhejiang University, Hangzhou 310058, China; 5 Ministry of Agriculture Key Lab of Molecular Biology of Crop Pathogens and Insects, Zhejiang University, Hangzhou 310058, China; 6 Zhejiang Provincial Key Laboratory of Biology of Crop Pathogens and Insects, Zhejiang University, Hangzhou 310058, China

**Keywords:** base composition, codon usage, evolutionary rate, gene rearrangements, phylogeny

## Abstract

Cynipoidea is a medium-sized superfamily of Hymenoptera with diverse lifestyles. In this study, 16 mitochondrial genomes were newly sequenced, 11 of which were the first obtained mitochondrial genomes in the family Liopteridae and four subfamilies (Anacharitinae, Aspicerinae, Figitinae, and Parnipinae) of Figitidae. All of the newly sequenced mitogenomes have unique rearrangement types within Cynipoidea, whereas some gene patterns are conserved in several groups. *nad5*-*nad4*-*nad4L*-*nad6*-*cytb* was remotely inverted and two rRNA genes were translocated to *nad3* downstream in Ibaliidae and three subfamilies (Anacharitinae, Eucoilinae, and Parnipinae within Figitidae); two rRNA genes in Aspicerinae, Figitinae, and Liopteridae were remotely inverted to the *cytb*-*nad1* junction; *rrnL*-*rrnS* was translocated to the *cytb*-*nad1* junction in Cynipidae. Phylogenetic inference suggested that Figitidae was a polyphyletic group, while the Ibaliidae nested deep within Cynipoidea and was a sister-group to the Figitidae. These results will improve our understanding of the gene rearrangement of the mitogenomes and the phylogenetic relationships in the Cynipoidea.

## 1. Introduction

Cynipoidea is a medium-sized superfamily of Hymenoptera, including around 223 genera with 3200 species described worldwide [1,2]. It includes five generally accepted extant families, Austrocynipidae, Cynipidae, Figitidae, Ibaliidae, and Liopteridae [2,3]. Cynipoid wasps exhibit a wide range of lifestyles [1,4,5]. The most well-known members are phytophagous and easily observed as gall-formers, while the majority of the species are small parasitoids or hyperparasitoids. Although previous molecular and/or morphological studies contributed to elucidating the family or subfamily relationships within Cynipoidea [1,6,7], the phylogenetic relationships within the Cynipoidea are still unclear and need further study, especially the phylogenetic relationships within the family Figitidae and Cynipidae.

The typical insect mitochondrial genome is a circular molecule that is 14–19 kb in size and encodes 37 genes, including 13 protein-coding genes (PCGs), two ribosomal RNA (rRNA) genes, and 22 transfer RNA (tRNA) genes [8]. Mitogenomes have been widely used for phylogenetics, though with the limitation of a relatively high evolutionary rate and the presence of base composition bias [8,9,10]. Most mitogenomes of insects are highly conserved, possessing the ancestral mitogenome arrangement; however, hymenopteran mitogenomes show extremely high rates of genome rearrangements [11,12,13,14]. tRNA rearrangements are widespread in Hymenoptera, with at least one tRNA rearrangement found in every sequenced hymenopteran species. Gene rearrangements are usually confined to specific lineages, which can help with phylogenetic reconstruction at lower taxonomic levels, such as the subfamily level in Braconidae [13]. At present, the complete or partial mitogenomes of only seven cynipoid species are available in GenBank (https://www.ncbi.nlm.nih.gov/; accessed on 20 January 2022). The synapomorphic mitochondrial gene rearrangement characters and their phylogenetic utility could not be fully assessed due to limited taxon sampling in the early studies.

In this study, 16 mitogenomes of Cynipoidea were newly sequenced by next generation sequencing (NGS), and 11 of them were the first obtained mitogenomes in the family Liopteridae and four subfamilies (Anacharitinae, Aspicerinae, Figitinae, and Parnipinae) of Figitidae. The obtained information from the study will also facilitate future phylogenetic research of Cynipoidea. Furthermore, we analyzed the main features of the newly generated mitogenomes and those of other Cynipoidea species. We also analyzed gene rearrangement patterns. Finally, the phylogeny of Cynipoidea was reconstructed by combining the available mitogenomes.

## 2. Materials and Methods

### 2.1. Sample Identification and DNA Extraction

All 16 newly sequenced samples were identified based on the morphology of adults according to the taxonomic literature (Table S1). All specimens were initially preserved in 100% ethanol and then stored at 4 °C before DNA extraction. Whole genomic DNA was non-destructively extracted from every sample using the DNeasy tissue kit (Qiagen, Hilden, Germany), modified from previous studies [15,16]. Voucher specimens were deposited in the Institute of Insect Sciences, Zhejiang University (Voucher specimen numbers: ZJUH_20220001- ZJUH_20220016, Table S1).

### 2.2. Next-Generation Sequencing and Assembly

All libraries were constructed using the VAHTS^®^ Universal DNA Library Prep Kit. Whole-genome data were generated on the Illumina NovaSeq platform (Illumina, San Diego, CA, USA) with a PE150 strategy (2 × 150 base, paired-end reads).

More than 2 GB of raw data of each sample was obtained. The raw reads were checked by FastQC v0.11.9 [17], with adapter contamination trimmed by Trimmomatic [18]. The target mitochondrial reads were filtered out using BLAST v2.9.0+ (BLASTn, E-value cutoff 1 × 10^−5^) against a reference dataset of published Cynipoidea mitogenomes [19]. The mitochondrial reads were assembled by SPAdes v3.0 [20] and IDBA v1.1.3 [21] with default parameters, respectively. Two assemblies were then integrated with GENEIOUS v2020.0.5 (Biomatters Ltd., San Diego, CA, USA).

### 2.3. Mitochondrial Genome Annotation and Analysis

Assembled contigs were initially annotated using the MITOS web server (http://mitos.bioinf.uni-leipzig.de/index.py; accessed on 15 June 2021) [22]. The start and stop positions of 13 PCGs were adjusted manually and corrected by aligning published data of Cynipoidea species in GenBank. The putative tRNA genes were confirmed by the tRNAscan-SE search server with their homologs from related species [23]. The obtained mitogenomes were submitted to GenBank (Accession number: OM677820-OM677835, Table 1).

The nucleotide composition of all components and the relative synonymous codon usage (RSCU) of PCGs were estimated using MEGA 11.0 [24]. The base composition values (AT and GC-skews) were calculated using the following formulas: AT-skew = (A − T)/(A + T) and GC-skew = (G − C)/(G + C) [25]. The numbers of the synonymous substitutions (Ks) and non-synonymous substitutions (Ka), and the ratios of Ka/Ks for each PCG were calculated in the DnaSP 6.0 [26]. The gene rearrangement of all protein-coding genes, all tRNAs, and two rRNAs in the 20 cynipoid mitogenomes were analyzed by comparison with the ancestral mitogenomes and with each other.

### 2.4. Phylogenetic Analysis

A total of 20 mitogenomes representing the superfamily Cynipoidea, including 16 newly obtained taxa, were used for phylogenetic analyses. Two species from Platygastridae, *Platygaster* sp. and *Trissolcus basalis* were used as outgroups (Table 1). The PCGs were realigned using the G-INS-i algorithm implemented in MAFFT v7.464 [27]. Bayesian inference analysis (BI) was conducted with MrBayes v3.2.7a [28] using the most optimal partition schemes and best model schemes (Table S2) acquired by PartitionFinder v1.1.1 [29]. Four independent Markov chains were run for 100 million generations, with tree sampling occurring every 1000 generations and a burn-in of 25% of the trees. The stationarity of the run was assessed by Tracer v1.7. (ESS values > 200) [30]. Maximum likelihood (ML) analysis was performed with RAxML-HPC2 v8.2.12 [31] under the GTRGAMMA model. A total of 200 runs for different individual partitions were conducted with 1000 bootstrap replicates.

## 3. Results and Discussion

### 3.1. General Features of Mitochondrial Genomes

**Figure 1:**
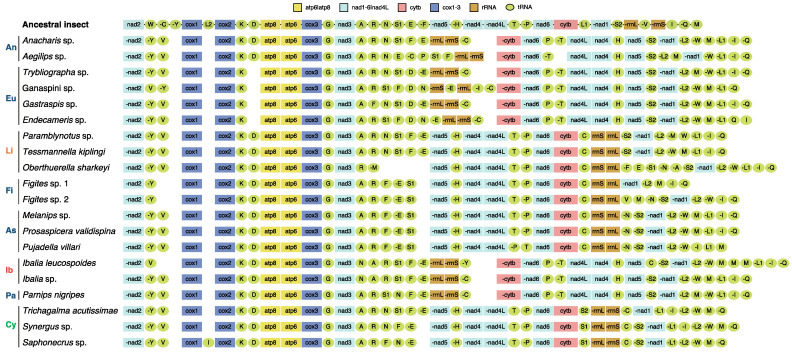
Mitogenomic architecture of the Cynipoidea referenced with the ancestral insect mitochondrial genome. An, Anacharitinae; Eu, Eucoilinae; Li, Liopteridae; Fi, Figitinae; As, Aspicerinae; Ib, Ibaliidae; Pa, Parnipinae; Cy, Cynipidae. Families are shown in different colors.

We obtained 16 new mitogenomes from the taxa of Cynipoidea. The 37 typical genes were identified in each of the newly sequenced mitogenomes except for *Trybliographa* sp. (missing *trnF*), *Figites* sp. 1 (missing *trnW*, *trnN*, *trnL1*, *trnS2*, and *trnV*), *Figites* sp. 2 (missing *trnL1*), and *Pujadella villari* (missing *trnQ*) (Figure 1). The complete control region (CR) of all the species failed to be assembled, possibly due to low similarity between reference and high A and T contents, common in insect mitogenomes, especially in Hymenoptera [19].

The A + T content for the sequenced region of the mitogenomes in the Cynipoidea ranged from 79.36% (*Trybliographa* sp.) to 87.01% (*Paramblynotus* sp.) (Table 2). There was no significant difference in base composition among the species of Cynipoidea. Relative high A + T content in the mitogenomes is not unusual in Hymenoptera compared with other orders [32].

### 3.2. Base Composition, Codon Usage, and Evolutionary Rate

All 13 PCGs were identified in the newly generated mitogenomes, with sizes ranging from 10,986 bp (*Figites* sp. 1) to 11,381 bp (*Anacharis* sp.). The entire A + T content of all the PCGs ranged from 76.81% (*Pu. villari*) to 85.66% (*Ibalia* sp.) (Table 2). The AT-skew in all Cynipoidea mitogenomes was negative. The GC-skew in Anacharitinae, Eucoilinae, and Liopteridae was also negative, whereas *Paramblynotus* sp. in Lio-pteridae was positive (Table 2), which is an unusual feature of cynipoid mitogenomes. Two rRNA genes (*rrnS* and *rrnL*) were identified in all mitogenomes. The length of *rrnS* ranged from 792 bp (*Aegilips* sp.) to 879 bp (*Endecameris* sp.), and the size of *rrnL* ranged from 1231 bp (*Ganaspini* sp.) to 1464 bp (*Pu. villari*) (Table S3).

**Figure 2:**
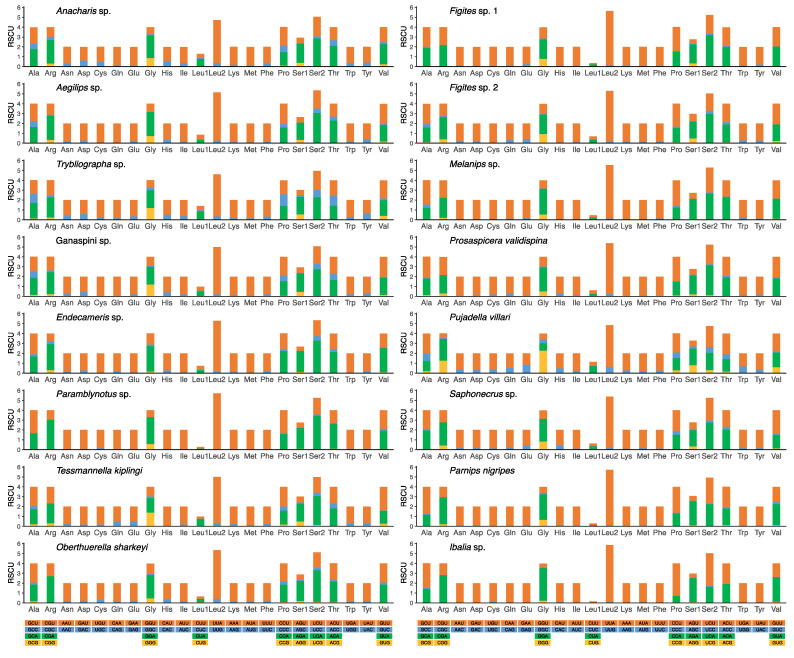
Relative synonymous codon usage (RSCU) of 16 cynipoid mitogenomes. Codon families are provided on the X-axis along with the different combinations of synonymous codons that code for that amino acid. RSCU is defined on the Y-axis.

The relative synonymous codon usage (RSCU) values of all four subfamilies were plotted in Figure 2, and all possible synonymous codons of the 22 amino acids were present. A or T nucleotides were used with higher frequency in the third codon position than other nucleotides, and A was used more often than T. The four most frequently used codons—AUA (Met), AUU (Ile), UUA (Leu2), and UUU (Phe)—were observed (Figure 2, Table S4). These results are consistent with published mitogenomes of other wasps [33,34].

**Figure 3:**
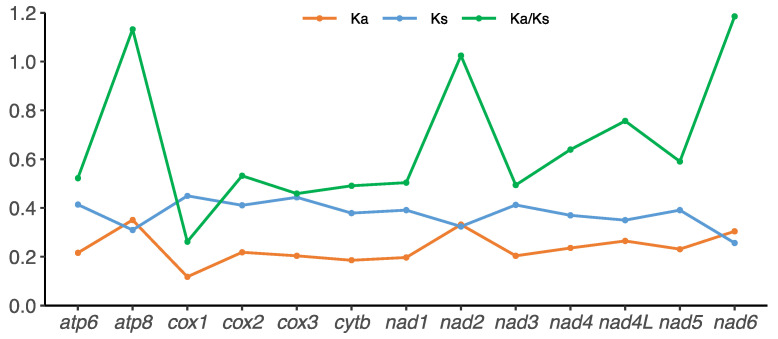
Non-synonymous (Ka), synonymous substitutional (Ks) rates and the ratios of Ka/Ks of 13 protein coding genes (PCGs) in mitochondrial genomes of all sequenced species in Cynipoidea.

Ka (nonsynonymous substitutions) and Ks (synonymous substitutions) are used as indicators of selective pressure [35]. The evolutionary rates (Ka, Ks, and Ka/Ks) of PCGs vary considerably among genes (Figure 3, Table S5). In Cynipoidea, the Ka values of 13 PCGs ranged from 0.1177 (*cox1*) to 0.3506 (*atp8*), and Ks values ranged from 0.2566 (*nad6*) to 0.4494 (*cox1*). The average Ka/Ks ratios were estimated to investigate the evolutionary rates of Cynipoidea PCGs. Ratios ranged from 0.2619 (*cox1*) to 1.1850 (*nad6*). The genes *nad2*, *atp8*, and *nad6* > 1, indicating that they evolved at a faster rate in Cynipoidea. This result was similar to that of the Apoidea [36] and Ichneumonidae [37] in Hymenoptera.

### 3.3. Gene Rearrangements

Compared with the putative ancestral mitogenome of insects, the mitogenomes of all the Cypoidea in this study are extremely variable, and all PCGs, tRNAs, and rRNAs had various degrees of rearrangement (Figure 2).

Gene *nad2* was locally inverted in all Cynipoidea species, as previously observed in two Chalcidoidea species, *Megaphragma amalphitanum* and *Eurytoma* sp. [38]. In addition, a large block, “*nad5*-*nad4*-*nad4L*-*nad6*-*cytb*”, was remotely inverted in Ibaliidae and three subfamilies (Anacharitinae, Eucoilinae, and Parnipinae) within Figitidae, which is a unique gene rearrangement pattern in Cynipoidea and had not been observed in other Hymenoptera mitogenomes.

Two rRNA genes were translocated to *nad3* downstream in Ibaliidae and three subfamilies (Anacharitinae, Eucoilinae, and Parnipinae) within Figitidae, which is consistent with the large block PCGs rearrangement mentioned above. In addition, the two rRNA genes in Aspicerinae, Figitinae, and Liopteridae was remotely inverted to the *cytb*-*nad1* junction. In Cynipidae, *rrnL*-*rrnS* was translocated to the *cytb*-*nad1* junction.

The tRNA genes exhibited an extremely diverse rearrangement in Cynipoidea. Except for the stable positions of three tRNA genes (*trnK*, *trnG*, and *trnH*), the other tRNA genes were more or less rearranged. The hot spots of rearrangement were concentrated in two tRNA clusters, *trnI*-*trnQ*-*trnM* and *trnA*-*trnR*-*trnN*-*trnS1*-*trnE*-*trnF*. The two tRNA clusters were rearranged to varying degrees in all species. Patterns of tRNA rearrangement were conserved in several groups. *trnD* was relatively stable and only rearranged in Eucoilinae. In Figitinae and Aspicerinae, the tRNA cluster *trnA*-*trnR*-*trnN*-*trnS1*-*trnE*-*trnF* was shuffled into *trnA*-*trnR*-*trnF*-*trnE*-*trnS1*. In Cynipidae, *trnL1* was translocated from upstream to downstream of *nad1*, and the cluster *trnL2*-*trnW*-*trnM*-*trnQ* was upstream of nad2. In Liopteridae, Ibaliidae, and two subfamilies (Anacharitinae and Parnipinae) of Figitidae, the cluster *trnL1*-*trnI*-*trnQ* was translocated upstream of *nad2*.

In conclusion, our analyses indicate that the rearrangement types of all newly sequenced mitogenomes in this study are novel within Cynipoidea. The gene rearrangements in Cynipoidea are randomly distributed, although they are conserved in several groups.

### 3.4. Phylogenetic Analyses

**Figure 4:**
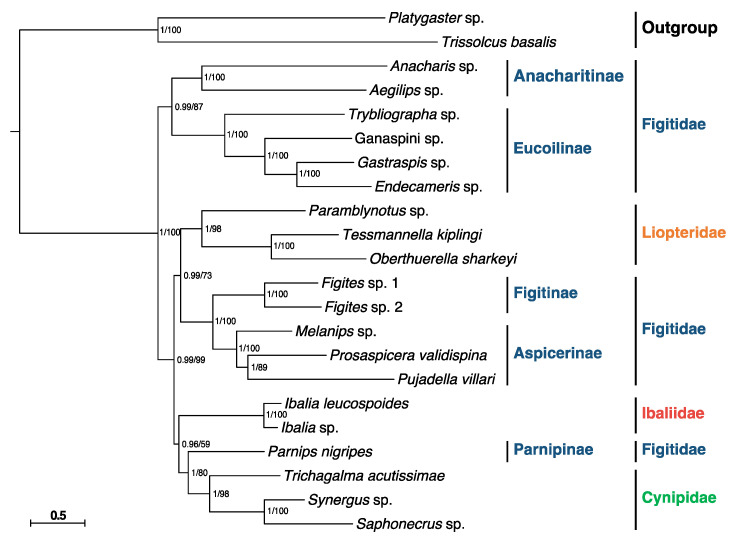
Phylogenetic analyses of Cynipoidea based on nucleotide datasets of 13 PCGs. The scale bar corresponds to the estimated number of substitutions per site. Numbers separated by a slash on the node are posterior probability (PP) and bootstrap value (BV).

Concerning the phylogenetic relationships of Cynipoidea, all analyses based on the PCGs matrix and two inference methods (BI and ML) generated congruent results with high nodal supports (Figure 4). The results indicate that the Figitidae is a polyphyletic group, consistent with the results based on UCEs [4]. The family Cynipidae is recovered as monophyletic by our data, as most previous studies assumed [1], but this contradicts the result of Blaimer et al. [4]. Adding more members to this family may help to show whether it is monophyletic or not. Ibaliidae was formerly thought to be an early-branching cynipoid in most studies [6,7]. However, Blaimer et al. proposed that Ibaliidae nested far inside Cynipoidea and was a sister-group to Figitidae [4]. Our research likewise came up with a similar result.

## 4. Conclusions

In this study, 16 Cynipoidea mitogenomes were newly obtained using the next-generation sequencing method, in which the mitogenomes of the family Liopteridae and four subfamilies Anacharitinae, Aspicerinae, Figitinae, and Parnipinae of Figitidae were first reported. The mitogenomes of all the Cypoidea in this research are highly variable. All of the newly sequenced mitogenomes in this study have unique rearrangement types within Cynipoidea. The gene rearrangements in Cynipoidea are randomly distributed, though there are gene patterns conserved in several groups, for instance, *nad5*-*nad4*-*nad4L*-*nad6*-*cytb* was remotely inverted and two rRNA genes were translocated to *nad3* downstream in Ibaliidae, and three subfamilies Anacharitinae, Eucoilinae, and Parnipinae within Figitidae; two rRNA genes in Aspicerinae, Figitinae, and Liopteridae were remotely inverted to the *cytb*-*nad1* junction; *rrnL*-*rrnS* was translocated to the *cytb*-*nad1* junction in Cynipidae. The BI and ML analysis showed consistent topology and indicated that Figitidae was a polyphyletic group and Ibaliidae nested far inside Cynipoidea and was a sister-group to Figitidae. Nevertheless, these results provide valuable information for understanding the evolution of Cynipoidea. Denser taxon sampling provides more accurate and comprehensive information for the further analysis of gene arrangement and the evolutionary history of Cynipoidea.

## Figures and Tables

**Table 1:** Information of mitochondrial genomes used in phylogenetic analysis.

Family	Subfamily	Species	Accession Number
Figitidae	Anacharitinae	*Anacharis* sp.	OM677820 *
Figitidae	Anacharitinae	*Aegilips* sp.	OM677821 *
Figitidae	Aspicerinae	*Melanips* sp.	OM677822 *
Figitidae	Aspicerinae	*Prosaspicera validispina*	OM677823 *
Figitidae	Aspicerinae	*Pujadella villari*	OM677824 *
Figitidae	Parnipinae	*Parnips nigripes*	OM677835 *
Figitidae	Eucoilinae	*Gastraspis* sp.	MG923497
Figitidae	Eucoilinae	*Endecameris* sp.	OM677825 *
Figitidae	Eucoilinae	Ganaspini sp.	OM677826 *
Figitidae	Eucoilinae	*Trybliographa* sp.	OM677827 *
Figitidae	Figitinae	*Figites* sp. 1	OM677828 *
Figitidae	Figitinae	*Figites* sp. 2	OM677829 *
Ibaliidae		*Ibalia leucospoides*	KJ814197
Ibaliidae		*Ibalia* sp.	OM677830 *
Liopteridae		*Paramblynotus* sp.	OM677831 *
Liopteridae		*Oberthuerella sharkeyi*	OM677832 *
Liopteridae		*Tessmannella kiplingi*	OM677833 *
Cynipidae		*Trichagalma acutissimae*	MN928529
Cynipidae		*Synergus* sp.	MG923514
Cynipidae		*Saphonecrus* sp.	OM677834 *
**outgroup**			
Platygastridae		*Platygaster* sp.	MG923507
Platygastridae		*Trissolcus* basalis	JN903532

* Newly obtained in this study.

**Table 2:** Base composition of 16 mitochondrial genomes in Chalcidoidea.

Species	Whole Genome		Protein-Coding Genes			
	Length (bp)	A + T (%)	Length (bp)	A + T (%)	AT-Skew	GC-Skew
*Anacharis* sp.	18,513	80.57	11,381	78.67	−0.0560	−0.1112
*Aegilips* sp.	16,709	84.00	11,080	81.75	−0.0832	−0.0722
*Melanips* sp.	16,103	85.52	11,186	83.67	−0.1121	0.0323
*Pr. validispina*	15,938	84.27	11,136	82.88	−0.1075	0.0336
*Pu. villari*	16,650	79.51	11,148	76.81	−0.1241	0.0662
*Endecameris* sp.	16,234	84.95	11,133	83.26	−0.0946	−0.0687
Ganaspini sp.	17,078	82.62	11,183	80.81	−0.0891	−0.0596
*Trybliographa* sp.	16,034	79.36	11,123	76.91	−0.0878	−0.1340
*Figites* sp. 1	15,333	84.24	10,986	82.90	−0.0930	0.0495
*Figites* sp. 2	16,775	83.09	11,145	81.31	−0.0984	0.0302
*Ibalia* sp.	17,176	86.40	11,069	85.66	−0.1175	0.0422
*Paramblynotus* sp.	15,482	87.02	11,173	85.58	−0.1127	0.0130
*O. sharkeyi*	16,053	84.04	11,199	82.58	−0.1060	−0.0169
*Te. kiplingi*	15,724	83.41	11,154	81.12	−0.1021	−0.0028
*Saphonecrus* sp.	16,482	85.36	11,271	82.97	−0.1049	0.0083
*Pa. nigripes*	16,876	84.46	11,168	83.82	−0.1114	0.0515

## Data Availability

The new mitogenome assemblies and annotation data in this study have been submitted to the GenBank database under accession numbers: OM677820-OM677835.

## Supplementary Materials

The following supporting information can be downloaded at: https://www.mdpi.com/article/10.3390/genes13050914/s1, Table S1: Collection information of Cynipoidea species in this study; Table S2: The best schemes of partition and substation models selected in nucleotide dataset; Table S3: Length of tRNA and rRNA genes in the Cynipoidea mitogenomes; Table S4: Relative synonymous codon usage in 16 Cynipoidea mitogenomes; Table S5: Synonymous and nonsynonymous substitutional analysis of 13 protein coding genes.
