# Supplementary material for: Novel Gene Rearrangements in the Mitochondrial Genomes of Cynipoid Wasps (Hymenoptera: Cynipoidea)
Source: Genes (Basel). 2022 May 20;13(5):914. doi: 10.3390/genes13050914 (PMC9140913; doi:10.3390/genes13050914)
Supplement: Supplementary file 1 [file genes-13-00914-s001.zip › genes-1667681-supplementary.pdf]

Supplementary tables

Table S1 Information of Cynipoidea species in this study.

Voucher specimens	Species	Sex	Locality	Date	Collector/Collect type	Identification reference
ZJUH_20220001	*Anacharis* sp.	Male	Zhangjiakou, Hebei, China	21-AUG-2005	Ying Zhang	[1,2]
ZJUH_20220002	*Aegilips* sp.	Male	Shangrao, Jiangxi, China	07-AUG-2015	Ning Mao	[1,2]
ZJUH_20220003	*Melanips* sp.	Male	Linzhi, Tibet, China	09-AUG-2019	Xiaofei Li	[1,2]
ZJUH_20220004	*Prosaspicera validispina*	Female	Linzhi, Tibet, China	16-AUG-2019	Zekai Li	[1-3]
ZJUH_20220005	*Pujadella villari*	Female	Linzhi, Tibet, China	16-AUG-2019	Zekai Li	[1,4]
ZJUH_20220006	*Endecameris* sp.	Female	Tianmu Mountain, Zhejiang, China	29-AUG-2018	Zekai Li	[1,2]
ZJUH_20220007	Ganaspini sp.	Male	Linzhi, Tibet, China	04-AUG-2019	Yellow trap	[1,2]
ZJUH_20220008	*Trybliographa* sp.	Male	Linzhi, Tibet, China	16-AUG-2019	Xingzhou Ma	[1,2]
ZJUH_20220009	*Figites* sp. 1	Male	Foping, Shaanxi, China	28-JUL-2013	Jiangli Tan	[1,2]
ZJUH_20220010	*Figites* sp. 2	Male	Sonoma, California, USA	07-SEP-2008	Peter H. Kerr	[1,2]
ZJUH_20220011	*Ibalia* sp.	Male	Shangrao, Jiangxi, China	07-AUG-2015	Shajiangyang Mao	[1,5]
ZJUH_20220012	*Paramblynotus* sp.	Female	Republic of the Congo	20-OCT-2008	Yves Braet, Michael J. Sharkey	[1,2]
ZJUH_20220013	*Oberthuerella sharkeyi*	Female	Republic of the Congo	20-OCT-2008	Yves Braet, Michael J. Sharkey	[1,2,6]
ZJUH_20220014	*Tessmannella kiplingi*	Female	Rikaze, Tiibet, China	01-AUG-2018	Yellow trap	[1,2,6]
ZJUH_20220015	*Saphonecrus* sp.	Female	Shangrao, Jiangxi, China	07-AUG-2015	Shajiangyang Mao	[1,7,8]
ZJUH_20220016	*Parnips nigripes*	Female	Madrid, Spain	19-MAR-2001	Jose Luis Nieves-Aldrey	[1,9]

Table S2 The best schemes of partition and substation models selected in nucleotide dataset.

Optimal partition	Best Model	Partition names
Partition 1	GTR+I+G	c1p2, c3p2, c2p2, a6p2
Partition 2	GTR+I+G	n4lp1, n5p1, n4p1, n3p1
Partition 3	GTR+I+G	cbp1, n1p1, c2p1, a6p1, c3p1
Partition 4	GTR+G	c1p3, c3p3, a6p3, c2p3, n3p3
Partition 5	GTR+I+G	n6p1, n2p1, a8p1
Partition 6	GTR+I+G	n4p2, n4lp2, n5p2, n3p2
Partition 7	GTR+G	n6p3, n1p3, cbp3
Partition 8	GTR+G	n5p3, n4lp3, n4p3
Partition 9	GTR+I+G	n1p2, cbp2
Partition 10	GTR+I+G	a8p2, n6p2, n2p2
Partition 11	GTR+I+G	c1p1
Partition 12	GTR+G	n2p3, a8p3

Table S3 Length of tRNA and rRNA genes in the Cynipoidea mitogenomes.

Species	trnW	trnC	trnY	trnL2	trnK	trnD	trnG	trnA	trnR	trnN	trnS1	trnE	trnF	trnH	trnT	trnP	trnL1	trnS2	trnV	trnI	trnQ	trnM	rrnS	rrnL
Anacharis sp.	72	63	71	71	72	70	70	69	69	70	62	69	66	74	67	72	71	69	73	71	67	69	824	1406
Aegilips sp.	67	68	67	65	69	67	66	57	65	65	61	72	64	65	64	72	67	69	66	68	68	68	792	1360
Melanips sp.	65	68	72	69	77	68	73	72	70	67	63	67	68	73	67	68	71	67	70	68	71	72	877	1418
Prosaspicera validispina	68	69	68	67	70	66	54	66	63	65	63	68	69	69	63	64	65	67	67	69	70	67	848	1360
Pujadella villari	67	69	72	69	69	70	68	68	71	67	64	68	65	53	67	66	68	68	70	66	-	68	858	1464
Endecameris sp.	72	65	65	65	72	74	69	66	69	65	60	68	66	71	65	77	67	67	66	65	71	63	879	1361
Ganaspini sp.	68	64	68	68	67	73	68	67	70	73	63	69	64	65	61	69	70	67	68	70	70	67	822	1231
Trybliographa sp.	67	66	65	66	69	69	66	66	66	67	60	65	-	68	65	67	68	68	66	69	67	67	812	1375
Figites sp. 1	-	72	69	68	68	68	68	64	67	-	65	66	65	64	65	69	-	-	-	65	70	67	811	1384
Figites sp. 2	66	67	70	69	69	68	66	66	68	69	63	66	67	66	67	66	-	68	70	66	69	70	813	1355
Ibalia sp.	69	64	66	67	72	69	67	65	67	68	64	68	63	65	65	69	74	68	59	67	67	75	811	1357
Paramblynotus sp.	66	63	64	66	64	64	67	66	70	67	60	69	64	66	63	65	69	66	69	65	69	68	824	1287
Oberthuerella sharkeyi	52	67	67	67	75	68	66	64	72	69	62	66	64	67	63	58	69	68	69	68	71	68	815	1362
Tessmannella kiplingi	69	63	68	67	69	65	69	69	67	66	62	58	62	53	62	55	69	64	71	70	70	67	793	1362
Saphonecrus sp.	68	69	65	70	69	66	68	70	69	69	64	67	67	69	64	66	72	68	68	69	70	68	844	1409
Parnips nigripes	70	67	67	67	70	68	66	65	65	66	64	67	65	69	64	66	54	68	66	69	70	70	821	1377

Table S4 Relative synonymous codon usage in 16 Cynipoidea mitogenomes.

	Anacharis sp.		*Aegilips* sp.		*Melanips* sp.		*Prosaspicera validispina*		*Pujadella villari*		*Endecameris* sp.		Ganaspini sp.		*Trybliographa* sp.		*Figites* sp. 1		*Figites* sp. 2		*Ibalia* sp.		*Paramblynotus* sp.		*Oberthuerella sharkeyi*		*Tessmannella kiplingi*		*Saphonecrus* sp.		*Parnips nigripes*	
Codon	Count	RSCU	Count	RSCU	Count	RSCU	Count	RSCU	Count	RSCU	Count	RSCU	Count	RSCU	Count	RSCU	Count	RSCU	Count	RSCU	Count	RSCU	Count	RSCU	Count	RSCU	Count	RSCU	Count	RSCU	Count	RSCU
UUU(F)	285	1.67	309	1.84	343	1.88	346	1.94	307	1.77	329	1.81	320	1.78	287	1.68	309	1.9	297	1.79	374	1.96	387	1.95	345	1.78	320	1.77	348	1.89	343	1.93
UUC(F)	57	0.33	26	0.16	22	0.12	10	0.06	39	0.23	34	0.19	39	0.22	55	0.32	17	0.1	35	0.21	8	0.04	10	0.05	42	0.22	42	0.23	20	0.11	12	0.07
UUA(L)	368	4.36	450	4.93	500	5.42	469	5.15	381	4.24	479	5.16	446	4.8	406	4.27	502	5.47	473	5.12	553	5.78	591	5.68	482	4.98	435	4.68	503	5.18	553	5.6
UUG(L)	30	0.36	20	0.22	11	0.12	21	0.23	55	0.61	11	0.12	19	0.2	31	0.33	16	0.17	16	0.17	7	0.07	3	0.03	36	0.37	30	0.32	20	0.21	12	0.12
CUU(L)	38	0.45	40	0.44	21	0.23	32	0.35	37	0.41	38	0.41	40	0.43	43	0.45	12	0.13	30	0.32	11	0.11	20	0.19	24	0.25	24	0.26	24	0.25	15	0.15
CUC(L)	5	0.06	9	0.1	3	0.03	3	0.03	3	0.03	3	0.03	5	0.05	10	0.11	1	0.01	3	0.03	0	0	0	0	0	0	3	0.03	3	0.03	1	0.01
CUA(L)	66	0.78	29	0.32	18	0.2	20	0.22	62	0.69	26	0.28	46	0.5	79	0.83	20	0.22	32	0.35	3	0.03	10	0.1	34	0.35	66	0.71	33	0.34	10	0.1
CUG(L)	0	0	0	0	0	0	1	0.01	1	0.01	0	0	1	0.01	1	0.01	0	0	0	0	0	0	0	0	5	0.05	0	0	0	0	2	0.02
AUU(I)	376	1.66	442	1.86	482	1.97	446	1.89	388	1.83	515	1.93	457	1.81	387	1.65	449	1.92	456	1.88	505	1.98	498	1.96	477	1.87	452	1.83	463	1.9	459	1.94
AUC(I)	76	0.34	33	0.14	7	0.03	25	0.11	36	0.17	19	0.07	49	0.19	82	0.35	18	0.08	29	0.12	6	0.02	9	0.04	32	0.13	41	0.17	24	0.1	15	0.06
AUA(M)	418	1.86	377	1.91	394	1.92	399	1.89	349	1.79	386	1.94	389	1.92	335	1.84	382	1.92	376	1.87	394	1.95	363	1.97	340	1.85	370	1.88	381	1.85	374	1.93
AUG(M)	31	0.14	18	0.09	16	0.08	23	0.11	42	0.21	11	0.06	17	0.08	29	0.16	16	0.08	26	0.13	10	0.05	6	0.03	28	0.15	24	0.12	32	0.15	14	0.07
GUU(V)	53	1.51	59	2.05	54	1.85	68	2.05	77	1.82	33	1.43	61	2.05	67	1.85	66	1.96	69	2.08	28	1.38	37	1.92	48	1.94	67	2.39	58	2.37	44	1.56
GUC(V)	8	0.23	3	0.1	1	0.03	5	0.15	5	0.12	1	0.04	2	0.07	7	0.19	1	0.03	1	0.03	1	0.05	3	0.16	6	0.24	2	0.07	4	0.16	6	0.21
GUA(V)	71	2.03	48	1.67	59	2.02	59	1.77	62	1.47	56	2.43	52	1.75	57	1.57	68	2.01	56	1.68	50	2.47	36	1.87	44	1.78	36	1.29	33	1.35	60	2.12
GUG(V)	8	0.23	5	0.17	3	0.1	1	0.03	25	0.59	2	0.09	4	0.13	14	0.39	0	0	7	0.21	2	0.1	1	0.05	1	0.04	7	0.25	3	0.12	3	0.11
UCU(S)	86	1.99	71	1.85	100	2.52	83	2	86	2.1	71	1.62	78	1.79	83	1.95	74	1.9	74	1.76	127	3.32	63	1.69	63	1.57	63	1.6	90	2.29	100	2.61
UCC(S)	10	0.23	17	0.44	5	0.13	4	0.1	24	0.59	20	0.46	24	0.55	32	0.75	6	0.15	12	0.29	4	0.1	5	0.13	10	0.25	13	0.33	9	0.23	4	0.1
UCA(S)	122	2.83	117	3.05	105	2.64	126	3.04	72	1.76	143	3.26	119	2.73	95	2.24	124	3.18	123	2.93	62	1.62	127	3.4	129	3.2	115	2.92	106	2.7	85	2.22
UCG(S)	0	0	0	0	0	0	4	0.1	12	0.29	0	0	0	0	1	0.02	0	0	2	0.05	0	0	1	0.03	4	0.1	5	0.13	1	0.03	0	0
CCU(P)	66	1.89	54	2.08	67	2.58	61	2.44	55	1.93	40	1.58	49	1.9	44	1.41	64	2.42	62	2.34	80	3.23	57	2.3	48	1.83	51	2	56	2.15	69	2.65
CCC(P)	23	0.66	9	0.35	5	0.19	2	0.08	16	0.56	5	0.2	15	0.58	37	1.18	2	0.08	4	0.15	2	0.08	3	0.12	9	0.34	11	0.43	9	0.35	1	0.04
CCA(P)	48	1.37	40	1.54	32	1.23	35	1.4	35	1.23	55	2.18	38	1.48	42	1.34	39	1.47	38	1.43	16	0.65	39	1.58	46	1.75	36	1.41	39	1.5	33	1.27
CCG(P)	3	0.09	1	0.04	0	0	2	0.08	8	0.28	1	0.04	1	0.04	2	0.06	1	0.04	2	0.08	1	0.04	0	0	2	0.08	4	0.16	0	0	1	0.04
ACU(T)	59	1.33	54	1.33	52	1.65	60	1.94	76	2.11	47	1.59	61	1.72	75	1.55	61	1.92	64	1.88	57	2.07	35	1.32	50	1.67	51	1.7	54	1.7	65	2.1
ACC(T)	25	0.56	16	0.4	2	0.06	5	0.16	17	0.47	8	0.27	22	0.62	50	1.04	3	0.09	8	0.24	1	0.04	2	0.08	6	0.2	15	0.5	9	0.28	4	0.13
ACA(T)	91	2.06	91	2.25	71	2.25	58	1.87	46	1.28	63	2.14	59	1.66	66	1.37	63	1.98	61	1.79	51	1.85	67	2.53	62	2.07	53	1.77	64	2.02	52	1.68
ACG(T)	2	0.05	1	0.02	1	0.03	1	0.03	5	0.14	0	0	0	0	2	0.04	0	0	3	0.09	1	0.04	2	0.08	2	0.07	1	0.03	0	0	3	0.1
GCU(A)	30	1.71	31	1.8	44	2.48	39	2.11	43	2.02	35	2.09	24	1.5	22	1.38	33	2	36	2.03	39	2.48	34	2.34	29	1.97	31	1.97	26	1.93	54	2.73
GCC(A)	9	0.51	10	0.58	6	0.34	2	0.11	16	0.75	4	0.24	10	0.63	15	0.94	2	0.12	7	0.39	2	0.13	0	0	3	0.2	5	0.32	2	0.15	2	0.1
GCA(A)	31	1.77	27	1.57	20	1.13	31	1.68	22	1.04	28	1.67	28	1.75	25	1.56	30	1.82	26	1.46	22	1.4	24	1.66	25	1.69	24	1.52	26	1.93	23	1.16
GCG(A)	0	0	1	0.06	1	0.06	2	0.11	4	0.19	0	0	2	0.13	2	0.13	1	0.06	2	0.11	0	0	0	0	2	0.14	3	0.19	0	0	0	0
UAU(Y)	162	1.62	170	1.63	206	1.89	191	1.82	178	1.66	190	1.83	148	1.71	118	1.37	187	1.79	179	1.76	210	1.94	208	1.93	197	1.82	187	1.72	199	1.82	218	1.95
UAC(Y)	38	0.38	38	0.37	12	0.11	19	0.18	37	0.34	18	0.17	25	0.29	54	0.63	22	0.21	24	0.24	7	0.06	7	0.07	20	0.18	31	0.28	20	0.18	6	0.05
CAU(H)	48	1.45	49	1.63	63	1.97	52	1.73	49	1.53	50	1.72	49	1.61	48	1.5	60	1.9	61	1.94	54	1.93	60	1.94	51	1.67	59	1.87	48	1.55	58	1.9
CAC(H)	18	0.55	11	0.37	1	0.03	8	0.27	15	0.47	8	0.28	12	0.39	16	0.5	3	0.1	2	0.06	2	0.07	2	0.06	10	0.33	4	0.13	14	0.45	3	0.1
CAA(Q)	47	1.84	52	1.96	45	1.91	39	1.73	35	1.49	48	1.81	52	1.96	52	1.82	47	1.88	41	1.67	47	1.96	52	2	49	1.85	43	1.54	43	1.79	52	1.96

CAG(Q)	4	0.16	1	0.04	2	0.09	6	0.27	12	0.51	5	0.19	1	0.04	5	0.18	3	0.12	8	0.33	1	0.04	0	0	4	0.15	13	0.46	5	0.21	1	0.04
AAU(N)	222	1.71	275	1.82	265	1.96	263	1.93	203	1.59	242	1.85	216	1.72	181	1.58	258	1.89	232	1.8	277	1.99	299	1.97	244	1.83	259	1.77	262	1.83	265	1.91
AAC(N)	37	0.29	27	0.18	6	0.04	10	0.07	52	0.41	20	0.15	35	0.28	48	0.42	15	0.11	26	0.2	2	0.01	5	0.03	23	0.17	34	0.23	24	0.17	12	0.09
AAA(K)	137	1.81	133	1.89	139	1.93	136	1.96	119	1.72	128	1.91	147	1.87	128	1.83	137	1.89	140	1.85	148	1.97	143	1.97	148	1.85	136	1.79	154	1.93	138	1.97
AAG(K)	14	0.19	8	0.11	5	0.07	3	0.04	19	0.28	6	0.09	10	0.13	12	0.17	8	0.11	11	0.15	2	0.03	2	0.03	12	0.15	16	0.21	6	0.08	2	0.03
GAU(D)	40	1.4	55	1.75	57	2	54	1.89	51	1.67	49	1.75	41	1.55	39	1.42	52	1.93	50	1.82	49	2	55	1.96	52	1.82	49	1.85	53	1.83	51	1.89
GAC(D)	17	0.6	8	0.25	0	0	3	0.11	10	0.33	7	0.25	12	0.45	16	0.58	2	0.07	5	0.18	0	0	1	0.04	5	0.18	4	0.15	5	0.17	3	0.11
GAA(E)	68	1.89	53	1.8	61	1.82	64	1.91	37	1.17	70	1.94	67	1.81	54	1.66	64	1.78	53	1.61	68	2	61	1.97	61	1.82	47	1.52	55	1.59	65	1.91
GAG(E)	4	0.11	6	0.2	6	0.18	3	0.09	26	0.83	2	0.06	7	0.19	11	0.34	8	0.22	13	0.39	0	0	1	0.03	6	0.18	15	0.48	14	0.41	3	0.09
UGU(C)	23	1.53	28	1.93	23	1.92	21	1.83	23	1.64	19	2	24	2	22	1.76	23	1.92	25	2	25	1.92	25	1.85	24	1.71	23	1.84	29	1.87	30	2
UGC(C)	7	0.47	1	0.07	1	0.08	2	0.17	5	0.36	0	0	0	0	3	0.24	1	0.08	0	0	1	0.08	2	0.15	4	0.29	2	0.16	2	0.13	0	0
UGA(W)	70	1.65	73	1.85	70	1.77	73	1.87	54	1.32	79	1.95	70	1.79	68	1.74	71	1.87	67	1.72	75	1.97	70	1.92	69	1.89	63	1.7	68	1.84	76	1.95
UGG(W)	15	0.35	6	0.15	9	0.23	5	0.13	28	0.68	2	0.05	8	0.21	10	0.26	5	0.13	11	0.28	1	0.03	3	0.08	4	0.11	11	0.3	6	0.16	2	0.05
CGU(R)	10	1	11	1.13	18	1.71	19	1.85	5	0.48	8	0.8	14	1.37	15	1.54	18	1.85	13	1.27	12	1.2	10	0.98	14	1.3	18	1.71	12	1.23	11	1.07
CGC(R)	3	0.3	1	0.1	1	0.1	0	0	1	0.1	3	0.3	2	0.2	2	0.21	0	0	1	0.1	0	0	0	0	0	0	0	0	0	0	0	0
CGA(R)	24	2.4	24	2.46	21	2	19	1.85	23	2.19	26	2.6	23	2.24	20	2.05	21	2.15	23	2.24	28	2.8	31	3.02	29	2.7	21	2	23	2.36	28	2.73
CGG(R)	3	0.3	3	0.31	2	0.19	3	0.29	13	1.24	3	0.3	2	0.2	2	0.21	0	0	4	0.39	0	0	0	0	0	0	3	0.29	4	0.41	2	0.2
AGU(S)	26	0.6	22	0.57	24	0.6	25	0.6	25	0.61	18	0.41	25	0.57	24	0.56	19	0.49	32	0.76	16	0.42	19	0.51	23	0.57	23	0.58	28	0.71	19	0.5
AGC(S)	1	0.02	0	0	0	0	2	0.05	9	0.22	1	0.02	2	0.05	7	0.16	2	0.05	1	0.02	1	0.03	2	0.05	4	0.1	6	0.15	3	0.08	1	0.03
AGA(S)	84	1.95	68	1.77	81	2.04	80	1.93	68	1.66	93	2.12	81	1.86	75	1.76	75	1.92	72	1.71	93	2.43	79	2.11	82	2.04	71	1.8	65	1.66	94	2.46
AGG(S)	16	0.37	12	0.31	3	0.08	8	0.19	32	0.78	5	0.11	20	0.46	23	0.54	12	0.31	20	0.48	3	0.08	3	0.08	7	0.17	19	0.48	12	0.31	3	0.08
GGU(G)	27	0.73	29	0.84	31	0.83	36	0.97	27	0.64	38	1.13	34	0.93	30	0.75	45	1.15	40	1.01	15	0.43	24	0.69	39	1.08	37	0.99	33	0.87	24	0.64
GGC(G)	4	0.11	1	0.03	2	0.05	4	0.11	15	0.36	6	0.18	3	0.08	11	0.28	4	0.1	4	0.1	1	0.03	1	0.03	3	0.08	6	0.16	0	0	4	0.11
GGA(G)	86	2.32	83	2.41	98	2.61	90	2.43	31	0.74	86	2.55	66	1.81	72	1.8	77	1.97	78	1.97	115	3.33	95	2.73	86	2.39	55	1.48	87	2.3	97	2.6
GGG(G)	31	0.84	25	0.72	19	0.51	18	0.49	95	2.26	5	0.15	43	1.18	47	1.18	30	0.77	36	0.91	7	0.2	19	0.55	16	0.44	51	1.37	31	0.82	24	0.64

Table S5 Synonymous and nonsynonymous substitutional analysis of 13 proteincoding genes.

Genes	Ka	Ks	Ka/Ks
atp6	0.2160	0.4137	0.5221
atp8	0.3506	0.3096	1.1322
cox1	0.1177	0.4494	0.2619
cox2	0.2184	0.4107	0.5318
cox3	0.2036	0.4434	0.4592
cytb	0.1859	0.3787	0.4909
nad1	0.1972	0.3913	0.5039
nad2	0.3319	0.3241	1.0243
nad3	0.2038	0.4126	0.4940
nad4	0.2362	0.3696	0.6391
nad4L	0.2648	0.3498	0.7568
nad5	0.2309	0.3909	0.5908
nad6	0.3041	0.2566	1.1850

References

1. Buffington, M.L.; Forshage, M.; Liljeblad, J.; Tang, C.T.; van Noort, S. World Cynipoidea (Hymenoptera): a key to higher-level groups. *Insect Syst. Divers.* **2020**, *4*, doi:10.1093/isd/ixaa003.
2. van Noort, S.; Buffington, M.L.; Forshage, M. Afrotropical Cynipoidea (Hymenoptera). *Zookeys* **2015**, 1-176, doi:10.3897/zookeys.493.6353.
3. Wang, J.; Ros-Farre, P.; Wang, Y. Review of Prosaspicera Kieffer (Hymenoptera: Figitidae: Aspicerinae) with description of two new species in China. *Entomotaxonomia* **2014**, *36*, 67-74.
4. Ros-Farre, P. Pujadella Ros-Farre, a new genus from the oriental region, with a description of two new species (Hymenoptera : Figitidae : Aspicerinae). *Zoological Studies* **2007**, *46*, 168-175.
5. Nordlander, G.; Liu, Z.W.; Ronquist, F. Phylogeny and historical biogeography of the cynipoid wasp family Ibaliidae (Hymenoptera). *Syst. Entomol.* **1996**, *21*, 151-165, doi:10.1046/j.1365-3113.1996.d01-4.x.
6. Buffington, M.L.; van Noort, S. Revision of the Afrotropical Oberthuerellinae (Cynipoidea, Liopteridae). *Zookeys* **2012**, 1-154, doi:10.3897/zookeys.202.2136.
7. Schweger, S.; Melika, G.; Tang, C.T.; Yang, M.M.; Stone, G.N.; Nicholls, J.A.; Sinclair, F.; Hearn, J.; Bozso, M.; Penzes, Z. New species of cynipid inquilines of the genus Saphonecrus (Hymenoptera: Cynipidae: Synergini) from the Eastern Palaearctic, with a re-appraisal of known species world-wide. *Zootaxa* **2015**, *4054*, 1-84, doi:10.11646/zootaxa.3999.4.1.
8. Wang, Y.P.; Chen, X.X.; Pujade-Villar, J.; Wu, H.; He, J.H. The genus Saphonecrus Dalla Torre et Kieffer, 1910 (Hymenoptera: Cynipidae) in China, with descriptions of two new species. *Biologia* **2010**, *65*, 1034-1039, doi:10.2478/s11756-010-0120-9.
9. Ronquist, F.; Nieves-Aldrey, J.L. A new subfamily of Figitidae (Hymenoptera, Cynipoidea). *Zoological Journal of the Linnean Society* **2001**, *133*, 483-494, doi:10.1006/zjls.2001.0302.
